# Accelerated Weathering Increases the Release of Toxic Leachates from Microplastic Particles as Demonstrated through Altered Toxicity to the Green Algae *Raphidocelis subcapitata*

**DOI:** 10.3390/toxics9080185

**Published:** 2021-08-05

**Authors:** Márta Simon, Nanna B. Hartmann, Jes Vollertsen

**Affiliations:** 1 Department of the Built Environment, Aalborg University, Thomas Manns vej 23, 9220 Aalborg, Denmark; jesvollertsen@build.aau.dk; 2 Department of Environmental Engineering, Technical University of Denmark, Bygningstorvet 115, 2800 Kgs. Lyngby, Denmark; nibh@env.dtu.dk

**Keywords:** microplastic, weathering, *Raphidocelis subcapitata*, ecotoxicity

## Abstract

Studies that evaluate the impact of microplastic particles (MPs) often apply particles of pristine material. However, MPs are affected by various abiotic and biotic processes in the environment that possibly modify their physical and chemical characteristics, which might then result in their altered toxic effect. This study evaluated the consequence of weathering on the release of toxic leachates from microplastics. MPs derived from six marine antifouling paints, end-of-life tires, and unplasticised PVC were exposed to UV-C radiation to simulate weathering. Non-weathered and weathered MPs were leached in algae growth medium for 72 h to demonstrate additive release under freshwater conditions. The model organism, green algae *Raphidocelis subcapitata*, was exposed to the resulting leachates of both non-weathered and weathered MPs. The results of the growth inhibition tests showed that the leachates of weathered microparticles were more toxic than of the non-weathered material, which was reflected in their lower median effect concentration (EC_50_) values. Chemical analysis of the leachates revealed that the concentration of heavy metals was several times higher in the leachates of the weathered MPs compared to the non-weathered ones, which likely contributed to the increased toxicity. Our findings suggest including weathered microplastic particles in exposure studies due to their probably differing impact on biota from MPs of pristine materials.

## 1. Introduction

Microplastic pollution is perceived as a threat to the environment that potentially affects organisms at several trophic levels because of the particles’ small size [1]. Therefore, the recognition of the ubiquitous presence of microplastic particles (MPs) in the environment during the past decade has raised scientific and public concerns [2,3]. Microplastic is an umbrella term for particles measuring up to a few millimetres in their longest diameter, consisting of synthetic polymers or chemically modified natural materials, e.g., natural rubber in tires or resins in coating materials [4,5,6]. MPs encompass plastic particles manufactured in the given size range, termed primary MPs, as well as secondary MPs, which are defined as plastic fragments formed from larger items or surfaces through chemical breakdown processes and physical abrasion [7,8].

MPs’ possible biological impact on a wide range of organisms has drawn scientific attention towards these pollutants. For instance, MPs can cause physical harm to organisms by blocking the gastro-intestinal tract or possibly introducing associated toxic compounds to the environment, or directly to the organism upon ingestion, through the leaching of potentially toxic substances [1,9,10].

While the polymers constituting plastic materials are inert, the residues of catalysts and reactants from the production process, as well as additives, e.g., flame-retardants, softeners, and antioxidants, can be toxic to various organisms [11,12,13]. Lithner et al. (2011) [11] ranked plastic materials based on the hazard of their components that can be released during their production or end-of-life phase. However, this list focused on the risks associated with organic additives, while polymers also contain heavy metals and metalloids that can comprise a substantial fraction of the material [14,15]. These inorganic compounds containing, for example, lead, cadmium, zinc, and chromium may serve as pigments, biocides, reinforcing agents, or flame-retardants in plastic products [14,16,17,18]. MPs might serve as vectors for such compounds as they can easily be dispersed in the environment, owing to their small size [1,10]. The presence of inorganic substances has been reported in marine plastic litter and associated with the contamination of soil and sediment adjacent to a plastic waste recycling facility or boat maintenance site [15,17,19]. Nevertheless, heavy metal pollution from plastic waste has received insufficient scientific attention to date [14,15].

Numerous studies have documented the potential adverse effect of MPs on various organisms [20,21,22,23,24,25]. Nevertheless, the lack of standard testing protocols addressing the physical and chemical properties and applied concentration of the investigated MPs has led to laboratory investigations of limited ecological relevance [26]. Exposure studies often apply MPs of pristine materials in uniform shape and size in concentrations greatly exceeding their amount detected in various environmental compartments [27,28]. Such experimental designs fail to represent natural conditions that hinder the assessment of the ecological impact of MPs [26,27,28]. MPs occur in various forms and sizes that environmental forces continuously modify by the weathering of particles’ material, which induces changes in their chemical and physical properties [4,29]. UV exposure is the principal weathering factor of MPs, which can cause chemical modifications in materials [7,30]. The resulting alterations are indicated by, for instance, the loss of volatile compounds, a weakened polymer structure, and increased hydrophilicity in the exposed layers of the material [30,31]. Several studies have shown that the altered characteristics of the plastic material can influence its impact on and toxicity towards various organisms [4]. Weathering may enhance the mobility of chemically non-bound compounds, create toxic degradation products, or reduce toxicity by degrading hazardous substances or restricting their migration from the bulk of a material [32,33,34,35,36]. Moreover, exposure studies often focus on marine species, which has led to a limited understanding of the effect of MPs on freshwater organisms and ecosystems, despite the latter’s crucial ecological role with its direct connection to terrestrial habitats [37,38,39]. Such environments have been under disproportionately high anthropogenic stress that has resulted in immense biodiversity loss exceeding its decline in the marine environment [39].

The present study aimed to evaluate the impact of microparticle leachates derived from six commercial antifouling paints, end-of-life tires, and unplasticised PVC—materials which often contain a substantial fraction of heavy metals such as biocides, pigments, catalysts, and heat stabilisers [40,41,42]. Moreover, this study examined the effect of UV radiation through the accelerated weathering of microparticles on leachate toxicity. Thereby, the effects of pristine and weathered MPs were compared to assess the significance of conducting toxicity experiments with environmentally realistic MPs. The model organism investigated in this study was *Raphidocelis subcapitata,* a freshwater microalga species, to extend the scarce knowledge on the effect of MPs on the freshwater environment.

## 2. Materials and Methods

### 2.1. Materials

We used microparticles of six commercial marine antifouling paints, tire, and unplasticised polyvinylchloride (PVC) for the toxicity tests. The preparation of the paint particles is described in detail by Simon et al. (2021) [43]. Microparticles of five paints containing copper and zinc were mixed; this particle mixture is hereafter referred to as “paint mix.” The sixth paint (zinc paint) contained only zinc in the form of zinc oxide and zinc pyrithione. Rubber granulates (2–6 mm) derived from end-of-life tires, provided by Genan A/S, Denmark, were cryo-grinded with liquid nitrogen using an IKA A11 basic analytical mill to produce tire microparticles for the experiments; these are subsequently referred to as “TP.” Unplasticised PVC sheets (CV313250) were purchased from Goodfellow and cryo-grinded using the same analytical mill. All four generated microparticle samples were dry-sieved through 500 and 20 µm stainless steel meshes to obtain microparticles in the size range of 20–500 µm. The microparticles of each material were divided into two mass fractions, and one fraction was exposed to UV-C radiation of 253 nm in a BS-02 irradiation chamber (Opsytech Dr. Gröbel GmbH, Ettlingen, Germany). We evaluated the weathering of the materials in the preliminary tests by examining their Fourier-transform infrared (FTIR) spectra [43]. The zinc paint microparticles were subjected to accelerated weathering for seven days, the paint mix for 14, and the PVC and tire microparticles for 28 days. FTIR spectra of the tire particles could not be collected due to the inherent black carbon content of the material that absorbs the infrared light, resulting in uninterpretable spectra. Consequently, we chose to expose the tire particles to weathering for 28 days, which was the longest exposure time in our experiment.

### 2.2. Particle Characterisation

Particle size was measured as described in Simon et al. (2021) [43]. In brief, a FlowCam^®^ 8000 Series Dynamic Imaging Particle Analyzer (Fluid Imaging Technologies, Inc., Scarborough, MA, USA) and a Stereo Discovery v.8 stereomicroscope (Zeiss GmbH, Oberkochen, Germany) were used to obtain morphological data of the MPs. These techniques allowed for the quantification of particles measuring larger than 12 µm, both in their length and width, which were defined as the maximum and minimum Feret diameters, respectively.

### 2.3. Leaching

An amount of 300 mg microparticles was leached in 30 mL ISO 8692:2004 standard [44] algae growth medium resulting in a particle concentration of 10 g L^−1^. The media were prepared using ultrapure water (Merck Millipore, Darmstadt, Germany). The composition of the media is described in the Supplementary Materials (Table S1). The particle–media mixtures were agitated at 200 rpm at 20 °C in the dark on an orbital shaker. After 72 h of leaching, the liquid was filtered through a syringe filter with a 0.45 µm pore size to remove any microparticles. The UV-weathered TP leachate required an additional filtration step through a filter with a 0.22 µm pore size to remove fine particles. The leachates were either applied immediately for the toxicity tests or kept frozen at −20 °C until the test. Triplicate blanks were prepared with algae growth medium, agitated under the same conditions as the microparticles, then filtered and kept frozen for a couple of days until toxicity testing.

### 2.4. Growth Inhibition Tests

Toxicity tests were carried out according to Hartmann et al. (2010) [45] with the green algae *Raphidocelis subcapitata*, using a mini-scale test setup as first described by Arensberg et al. (1995) [46]. *R. subcapitata* was chosen as the model organism as it is suitable for testing the effect of substances on the growth inhibition of freshwater microalgae according to the OECD (2011) Test No. 201 guideline [47]. In 20 mL glass vials, 4 mL of algae suspensions (10^4^ algae cells mL^−1^ in the exponential growth phase) was exposed to a range of dilutions of microparticle leachates prepared with the growth media. The tests were conducted at a pH of 7.6–8.0, with typical control growth rates of 1.4–1.7 day^−1^ during the 48 h incubation. The test vials were agitated at 200 rpm in a temperature-controlled room (20 ± 1 °C), enabling mixing and CO_2_ diffusion. The samples were continuously illuminated with cold fluorescent light in the visible spectrum. Light intensity was 80–105 E m^−2^ s^−1^ in the wavelength range of 400–700 nm and was measured under the test vessels using an LI-COR light meter (modelLI-189) with an attached quantum sensor. Table 1 summarises the tested concentrations of the definitive tests expressed as the percentage of the concentrated leachate. All tests included triplicates of each tested concentration and five replicates of the control sample, which only contained algae and the growth medium. The pH of the leachates was adjusted with 0.1 M sodium hydroxide to 7.6–8.0 before the tests. A 0.4 mL sample was taken from each replicate after 48 h of incubation to extract chlorophyll and to measure its fluorescence, which is proportional to the algal biomass under the experimental conditions [48]. The rate of growth inhibition was calculated, and log-logistic concentration–response curves were fitted to estimate the effect concentration (EC) at 10%, 20%, and 50% growth inhibition using the drc package [49] of the software RStudio v.1.2.5. Toxicity tests with undiluted triplicate blank leachates were carried out as well to assess the quality of the media after the leachate preparation procedure. Furthermore, the effect concentrations of the single metal of copper, using CuSO_4_.5 H_2_O, and of zinc, applied as zinc chloride, were assessed similarly.

### 2.5. Chemical Analysis

Cadmium (Cd), chromium (Cr), copper (Cu), lead (Pb), and zinc (Zn) were measured in the concentrated leachates that were applied in the definitive tests and the blank samples, as the preliminary test revealed the presence of these heavy metals in the MP leachates in detectable concentrations. The measurements were carried out on the acidified leachates with an iCap 6000 inductively coupled plasma–optical emission spectrometer from Thermo Scientific using yttrium internal standard as described in Simon et al. (2021) [43].

## 3. Results

### 3.1. Particle Size Characteristics

**Figure 1:**
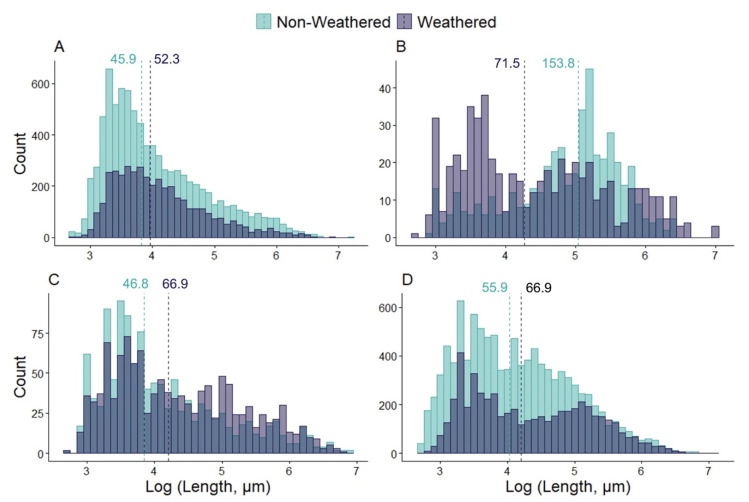
Distribution of the length of non-weathered and weathered paint mix (**A**), zinc paint (**B**), tire particle (TP) (**C**) and PVC (**D**) microparticles applied in the growth inhibition tests. The size of the bins was 0.1 on a logarithmic scale. The length is defined as the maximum Feret diameter, and the median values in micrometres are denoted.

Figure 1 illustrates the distribution of the length of the MPs applied in the growth inhibition tests. The median length of all weathered microparticles, except the zinc paint, was larger than that of the non-weathered MPs, despite UV exposure being expected to induce particle fragmentation that would have resulted in an increased number of small particles. The underlying reason for such a discrepancy could be that the size of the formed microparticle fragments was beyond the detection limit of the applied instrument and remained hidden from the analysis. A similar pattern was observed regarding the width of the particles demonstrated in Figure S1.

### 3.2. Toxicity of Leachates

The quality criteria of the growth inhibition tests were fulfilled, since the control growth rate measured between 1.4 and 1.7 day^−1^ and the most considerable pH change in the controls was 0.9 units, which was well below the accepted 1.5 unit change [44]. Table 2 shows the effect concentrations expressed as the percentage of the concentrated leachate and their 95% confidence intervals, while the concentration–response curves are found in the Supplementary Materials (Figure S2). All of the tested materials resulted in toxic leachates, and the UV-weathered variants were more toxic than the non-weathered at all toxicity levels. The blank leachates of algae growth media did not affect the growth of the algae (Table S2), meaning that the quality of the growth media did not deteriorate during the leaching procedure and storage. Therefore, it is safe to assume that the observed toxicity of the particle leachates is attributable to the compounds that leached from the MPs. Furthermore, the EC_50_ of the tested single copper and zinc ions was 32 and 80 µg L^−1^, respectively.

The leachates of the non-weathered paint mix, zinc paint, and tire microparticles exerted similar toxicities at the lower toxic levels, as their EC_10_ and EC_20_ values were comparable with overlapping confidence intervals. However, the zinc paint leachate was the most toxic among the non-weathered particles based on the EC_50_ values, followed by the paint mix, TP, and PVC leachates. The non-weathered PVC leachate was the least toxic among all of the tested materials.

The UV-weathered paint mix produced the most toxic leachate among all materials at all toxicity levels. The UV exposure of the PVC particles markedly increased the toxicity of their leachate concerning the non-weathered material. That is, while the PVC leachate was the least toxic at all toxicity levels among the non-weathered particles, the toxicity of the leachates from the UV-weathered PVC particles was similar to that of the leachates from the UV-weathered paint mix and exceeded that of the leachates from the UV-weathered TP and zinc paint. The toxicity of the leachates from UV-weathered tire and zinc paint microparticles were comparable at lower toxicity levels, but the EC_50_ of the zinc paint leachate was notably lower.

### 3.3. Elemental Composition of MP Leachates

The leachates of the paint mix microparticles contained zinc and copper in the highest concentrations, while zinc was present in the highest concentration in the leachate of the zinc paint microparticles (Table 3). The TP leachates also contained zinc in the highest concentrations, which likely originates from zinc oxide used as an activator in the vulcanisation process [42]. The leachate of the UV-weathered TP contained chromium, copper, and lead in detectable concentrations as well. The PVC leachate contained lead in the highest concentration, which is a thermal stabiliser added to the polymer as various lead salts [41,50]. The concentrations of heavy metals were markedly higher in the leachates of the UV-weathered particles than of the non-weathered material.

## 4. Discussion

The tested experimental conditions resulted in EC_50_ values of all MP leachates. The heavy metals that leached from the microparticles most likely contributed to the toxic effect of the leachates, as they have well-known antimicrobial properties [40,51,52]. Their role in the observed toxicity is highly plausible, as the concentrations of zinc and copper in the undiluted leachates of the paint and tire particles greatly exceeded the EC_50_ values of 80 and 32 µg L^−1^ obtained in our tests performed with zinc and copper, respectively. The concentration of lead in the PVC leachates was also higher than the 50 µg L^−1^ EC_50_ of *R. subcapitata* reported by Blinova (2004) [53]. Furthermore, the toxicity of the UV-weathered paint mix and the non-weathered PVC microparticles was consistent with the highest and lowest metal concentrations measured in the undiluted leachates.

However, heavy metal ions were likely not the only components in the leachates that induced toxic effects. That is, the nominal concentration of heavy metals corresponding to the EC_50_ dilution of the leachate indicates that their concentration was possibly lower than the EC_50_ of the single metals, except in the case of zinc in the non-exposed TP and paint mix leachate. For example, 3.9% of the concentrated non-weathered paint mix microparticle leachate caused 50% growth inhibition, which corresponds to an ion concentration of 3 µg L^−1^ of copper. This estimated concentration is markedly lower than the EC_50_ value of single copper ions of 32 µg L^−1^ determined in the present study. This is probably because the leachates were complex mixtures of heavy metals and released organic compounds, such as booster biocides, as well as organic components, e.g., additives and degradation products. The latter two can form lipophilic organometallic complexes with the released heavy metals in aqueous media, which increases the bioavailability of the metals [54]. Furthermore, the zinc pyrithione (ZnPT) booster biocide present in the zinc paint could be the cause of the higher toxicity of its microparticle leachate than of the non-weathered TP and paint mix leachate, as the total heavy metal concentration in the zinc paint leachate was distinctly lower. ZnPT is highly toxic to aquatic organisms (e.g., [55,56]) and *R. subcapitata*, reflected in its low EC_50_ of 28 µg L^−1^ [57].

Nevertheless, the obtained EC_50_ values for both the non-weathered and weathered TP leachates (1.2% and 4.7% leachate, respectively) was comparable to previously reported EC_50_ values of *R. subcapitata* of 0.5% and 0.93% leachate and 0.05–1.01 g L^−1^ particle concentration [58,59,60].

The UV-weathered paint mix microparticles resulted in the most toxic leachate. This finding is consistent with a previous study, which found that spent antifouling paint particles are toxic to the marine macroalga *Ulva lactuta* when applied in a few parts per million (ppm) [52]. The EC_50_ of the UV-weathered PVC leachate was comparable to the EC_50_ of 1.6% leachate reported by Capolupo et al. (2020), though the inorganic elemental composition of the two leachates differed. UV-weathered PVC leachate was more toxic than the UV-weathered zinc paint and TP leachates, despite that the total concentration of metals in the PVC, zinc paint, and the TP leachates were comparable.

In summary, the differences in EC_50_ values indicate that the UV-C weathered MPs rendered more toxic leachates than their non-weathered variants. The increased toxicity is likely partially associated with the higher heavy metal concentrations in the leachates of weathered MPs, which is possibly a consequence of UV-induced changes in the material, resulting in increased metal mobility [43]. Furthermore, co-leached organic substances from the matrix of the paint, tires, and PVC materials could form lipophilic complexes with heavy metals that could increase their bioavailability [40,54].

The results showed that the leachates of marine antifouling paint microparticles were toxic to a freshwater species, even though these materials were intended to prevent the growth of marine organisms. This finding is not surprising, due to the known general antimicrobial effects of zinc and copper, but highlights that further studies on MP ecotoxicological effects, focusing on the freshwater environment, are needed, as MPs can be equally deleterious to both marine and freshwater biota. Furthermore, the outcomes of the present study propose the inclusion of heavy metal controls in MP exposure studies, since MPs can contribute to an elevated concentration of these compounds in environmental matrices, as pointed out by Turner (2016) [14]. For instance, soil and sediment contamination has been associated with large plastic items of litter that consist of materials containing a notable fraction of heavy metals, such as the studied antifouling paint, tires, and unplasticised PVC, as well as plastic materials of electronic appliances and so on [14,33,61]. Therefore, investigating heavy metal release and impact on biota is of particular importance in the case of MPs derived from these types of materials.

The experimental design of the present study, namely, MP concentrations exceeding their measured environmental concentrations reported by Burns and Boxall (2018) [27], as well as applying an artificial leaching media, enabled a comparison of the impact of weathering on MP toxicity, as it minimised the effects of possible side reactions during leaching. However, heavy metals released into the aqueous phase undergo dilution and interact with dissolved organic and inorganic matter in the environment. Such interactions include, for example, chelation, complexation, precipitation, and subsequent sedimentation—processes which affect the bioavailability of the metals and the exposure of organisms [40,54]. Although these interactions should be taken into account for a realistic risk assessment of MPs, accounting for them was outside of the scope of the present study, which solely investigated the impact of weathering on MP toxicity.

## 5. Conclusions

The present study evaluated the toxic effect of microparticle leachates derived from various antifouling paint, end-of-life tire, and unplasticised PVC microparticles on a freshwater microalga species, *R. subcapitata*. It pointed to the fact that not only should the possible toxic effect of MPs themselves be considered, but also their potential for release of toxic leachates. Since a combination of particle- and chemically induced effects can be expected under natural conditions, both modes of action should be considered to conduct impact studies with ecological relevance. This approach requires extensive characterisation of MP leaching potential and identification of potentially toxic constituents. Nevertheless, when interpreting the results from such studies, care should be taken to distinguish between particle- and chemically induced effects, as has been recommended for nanomaterial testing [62]. Our results further demonstrated that weathering can significantly enhance the toxic ecological impact of the studied MPs, possibly through mobilising heavy metals in the materials. Consequently, conducting impact studies with weathered MPs might reflect the processes occurring under natural conditions in freshwater ecosystems, as well as potentially in the marine environment, better than MPs of pristine material.

## Figures and Tables

**Table 1:** The tested concentrations of the definitive tests expressed as the percentage of the concentrated leachate (% of concentrated leachate). Non-weathered refers to microparticles not exposed to UV irradiation, while UV-weathered refers to microparticles exposed to UV-C. The tested concentrations of the weathered microparticle leachates were lower than the non-weathered one, as the range-finding tests demonstrated higher toxicity of these to the algae.

Concentration (% of Concentrated Leachate)							
TP	TP	Paint Mix	Paint Mix	Zinc Paint	Zinc Paint	PVC	PVC
Non- Weathered	UV- Weathered	Non- Weathered	UV- Weathered	Non- Weathered	UV- Weathered	Non- Weathered	UV- Weathered
100	20	10	1	5	3.33	100	6.67
50	10	5	0.5	3.33	2.22	50	3.33
25	5	2.5	0.25	2.22	1.48	25	1.66
12.5	2.5	1.25	0.125	1.48	0.99	12.5	0.83
6.25	1.25	0.63	0.063	0.99	0.66	6.25	0.42
3.1	0.63				0.44		0.21
							0.1

**Table 2:** EC values expressed as % leachate, with the lower and upper confidence intervals of the EC values included. The EC values of single copper and zinc ions are expressed in micrograms per liter.

		EC_10_	EC_20_	EC_50_
Non-weathered	TP	1.7 (1.3–2.1)	2.5 (2.1–2.9)	4.7 (4.3–5.1)
	Paint mix	2.2 (1.8–2.6)	2.7 (2.3–3.1)	3.9 (3.3–4.4)
	Zinc paint	1.9 (1.8–2.0)	2.1 (2.0–2.2)	2.5 (2.3–2.6)
	PVC	8.8 (3.9–13.7)	13.5 (8.3–18.8)	28.5 (22.7–34.3)
UV-weathered	TP	0.4 (0.3–0.5)	0.6 (0.5–0.7)	1.2 (1.1–1.3)
	Paint mix	0.21 (0.18–0.24)	0.24 (0.22–0.27)	0.3 (0.26–0.37)
	Zinc paint	0.47 (0.41–0.52)	0.54 (0.50–0.58)	0.7 (0.66–0.73)
	PVC	0.21 (0.15–0.27)	0.31 (0.25–0.38)	0.6 (0.54–0.68)
Zn ion (µg L^−1^)		30 (15–43)	40 (27–57)	80 (66–95)
Cu ion (µg L^−1^)		17 (15–19)	21 (19–23)	32 (22–41)

**Table 3:** The blank-corrected concentrations of inorganic elements measured in the concentrated leachates. Total metal refers to the sum of metal concentrations. ND denotes non-detected concentrations.

		Concentration (µg L^−1^)					
		Cd	Cr	Cu	Pb	Zn	Total Metal
Non- weathered	TP	ND	ND	ND	ND	1841	1841
	Paint mix	ND	ND	78	0.48	2775	2854
	Zn paint	ND	ND	13	0.48	956	970
	PVC	ND	ND	2.5	113	80	195
UV- weathered	TP	ND	2.18	13	1.88	7187	7204
	Paint mix	ND	ND	2339	3.41	23,864	26,207
	Zn paint	1.8	ND	3.1	1.02	7253	7259
	PVC	1.4	ND	7.6	6630	52	6691

## Data Availability

Data is available upon request from the corresponding author.

## Supplementary Materials

The following are available online at https://www.mdpi.com/article/10.3390/toxics9080185/s1, Figure S1: Distribution of the width of non-weathered and weathered microparticles; Figure S2: Concentration–response profiles of particle leachates; Table S1: Composition of the algae growth medium; Table S2: Growth rates of blank samples.
