# Supplementary material for: Accelerated Weathering Increases the Release of Toxic Leachates from Microplastic Particles as Demonstrated through Altered Toxicity to the Green Algae Raphidocelis subcapitata
Source: Toxics. 2021 Aug 5;9(8):185. doi: 10.3390/toxics9080185 (PMC8402562; doi:10.3390/toxics9080185)
Supplement: Supplementary file 1 [file toxics-09-00185-s001.zip › toxics-1304736-supplementary.pdf]

toxics MDPI

Supplementary Materials: Accelerated Weathering Increases the Release of Toxic Leachates from Microplastic Particles as Demonstrated through Altered Toxicity to the Green Algae *Raphidocelis subcapitata*

Márta Simon, Nanna B. Hartmann and Jes Vollertsen

Table S1. Composition of the algae growth medium.

NH_4Cl	15	mg/L
$MgCl_2.6\ H_20$	12	mg/L
$CaCl_2.7\ H_20$	18	mg/L
$MgSO_4.7\ H_20$	15	mg/L
KH_2PO_4	1.6	mg/L
$FeCl_3.6\ H_20$	64	µg/L
Na_2EDTA	100	µg/L
H_3BO_4	185	µg/L
$MnCl_2.4\ H_2O$	415	µg/L
$ZnCl_2$	3	µg/L
$CoCl_2.6\ H_2O$	1.5	µg/L
$CuCl_2.2\ H_2O$	0.1	µg/L
$Na_2MoO_4.2\ H_2O$	7	µg/L
$NaHCO_3$	50	mg/L

Table S2. Growth rates of blank samples. The growth medium was agitated at 20 °C for 72 hours. Afterwards, the samples were filtered through a 0.45 µm syringe filter and kept frozen at −20 °C for a couple of days, prior to the growth inhibition tests.

Sample		Growth rate
Blank 1	1	1.17
	2	1.15
	3	1.15
Blank 2	1	1.22
	2	1.38
	3	1.32
Blank 3	1	1.26
	2	1.32
	3	1.31

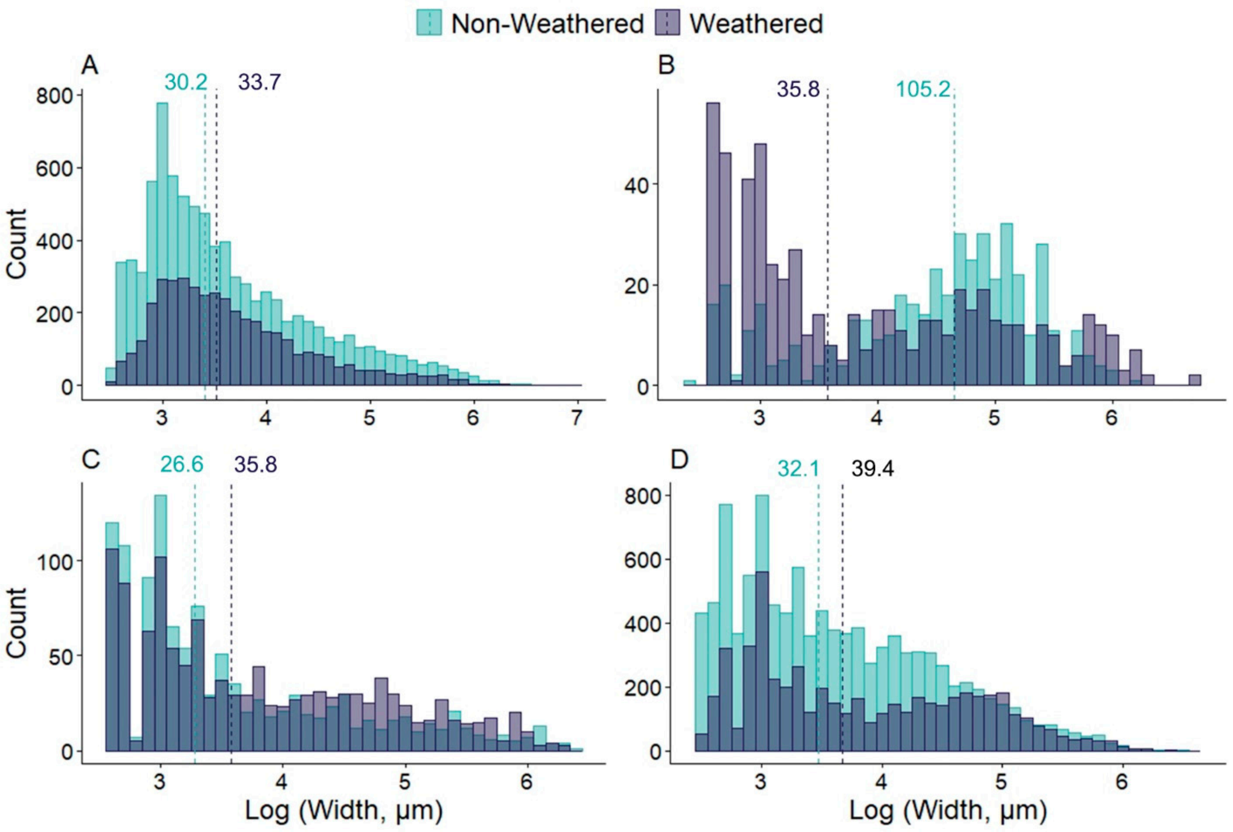

Figure S1. Distribution of the width of non-weathered and weathered paint mix (A), zinc paint (B), tire (C) and PVC (D) microparticles applied in the growth inhibition tests. The size of the bins was 0.1 on a logarithmic scale. The width is defined as the minimum Feret diameter, and the median values in micrometre are denoted.

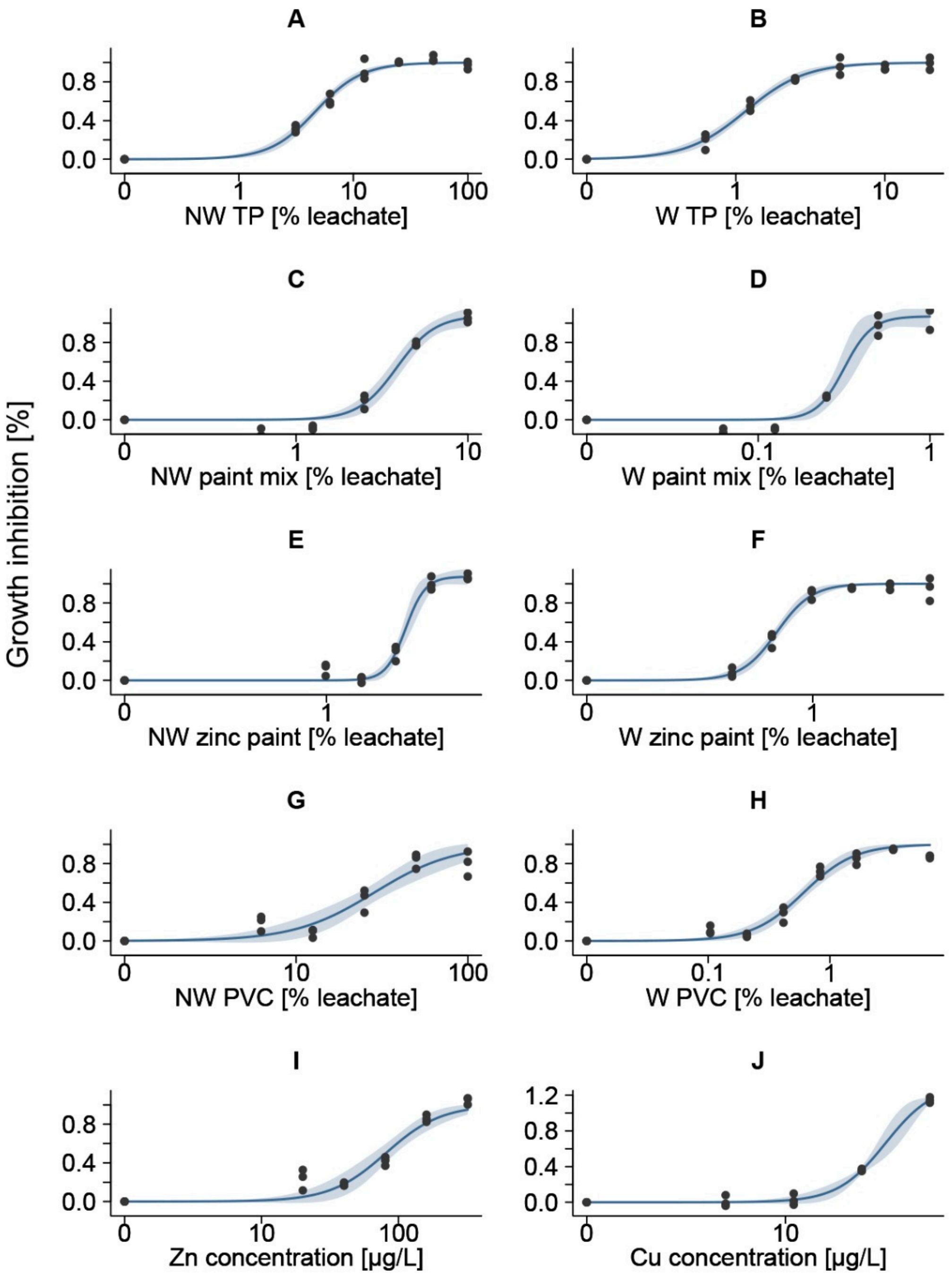

Figure S2. Log-logistic concentration-response profiles of particle leachates, including 95% confidence interval.
